# Supplementary material for: Recurrence rate of preterm birth and associated factors among women who delivered at Kilimanjaro Christian Medical Centre in Northern Tanzania: A registry based cohort study
Source: PLoS One. 2020 Sep 14;15(9):e0239037. doi: 10.1371/journal.pone.0239037 (PMC7489548; doi:10.1371/journal.pone.0239037)
Supplement: S1 Questionnaire — (DOCX) [file pone.0239037.s001.docx]

**Questionnaire**

**1 Basic information concerning mother**

| 1.1 Mothers date of birth : |  |  | |  |  | 1.2 Age: |  |  |  |  |  |  |
| --- | --- | --- | --- | --- | --- | --- | --- | --- | --- | --- | --- | --- |
|  |  |  | |  |  |  |  |  |  |  |  |  |
|  |  |  | |  |  |  |  |  |  |  |  |  |
| 1.3 Hospital number : |  |  | |  |  | 1.4 Birth number : | | |  |  |  |  |
| 1.5 Date of interview: |  |  | |  |  | 1.6 Date of admission: | | |  |  |  |  |
| **1.7 Referred for delivery:** | Yes | | | | | 1.8 If yes: **Referred from:** | | |  | 1 Home | |  |
|  | No | | | | |  |  |  |  | 2 | Regional hospital |  |
|  |  |  | |  |  |  |  |  |  | 3 | District hospital |  |
|  |  |  | |  |  |  |  |  |  | 4 other, specify: | |  |
| 1.9 Referred during labour: |  | 1 Admitted in labour | | | | 1.10 Reason for referral: | | |  |  |  |  |
|  |  |  |  |  |  |  |  |  |  |  |  |  |
|  |  | 2 Admitted before labour | | | |  |  |  |  |  |  |  |
|  | |  | |  |  |  |  |  | |  |  |  |
| 1.11 Official date of discharge: | |  | |  |  | 1.12 Date of leaving hospital: | | | |  |  |  |
|  |  |  | |  |  |  |  |  |  |  |  |  |
|  |  |  | |  |  |  |  |  | |  | |  |
| 1.13 Current residence |  | 1 Rural | | | | 1.14 Highest educational level: | | | | 1 None | |  |
|  |  | 2 Urban | | | |  |  |  |  | 2 | Primary (1-7) |  |
|  |  | 3 Semi urban | | | |  |  |  |  | 3 Secondary (8-11) | |  |
|  |  |  |  | |  |  |  |  |  | 4 | Higher (12+) |  |
| 1.15 Current occupation: |  | 1 Housewife | | | | 1.16 Current marital status: | | | | 1 Married | |  |
|  |  | 2 Farmer | | | |  |  |  |  | 2 | Single |  |
|  |  | 3 Service | | | |  |  |  |  | 3 Widowed | |  |
|  |  | 4 Business | | | |  |  |  |  | 4 Remarried | |  |
|  |  | 5 Professional | | | |  |  |  |  | 5 | Divorced |  |
|  |  | 6 Student | | | |  |  |  |  | 6 Polygamous family | |  |
|  |  | 7 Other, specify: | | |  |  |  |  |  |  |  |  |
|  |  |  | |  |  |  |  |  |  | | |  |
| 1.17 Mother's tribe: | 1 Chagga | | | | | 1.18 Religion: | | | 1 Catholic | | |  |
|  | 2 Pare | | | | |  |  |  | 2 Protestant | | |  |
|  | 3 Masai | | | | |  |  |  | 3 Muslim | | |  |
|  | 4 Other, specify: | | |  |  |  |  |  | 4 Others, specify: | | |  |
|  |  |  | |  |  |  |  |  |  |  |  |  |


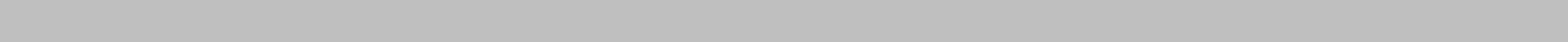
**2 Mothers health before and during present pregnancy**

| 2.1 Body weight (kg) | | | |  | | |  |  |  | | | 2.2 Body height (cm): | | | | |  | | |  |  | |  |  |  |
| --- | --- | --- | --- | --- | --- | --- | --- | --- | --- | --- | --- | --- | --- | --- | --- | --- | --- | --- | --- | --- | --- | --- | --- | --- | --- |
|  |  |  |  |  | | |  |  |  | | |  |  |  |  |  |  | | |  |  | |  |  |  |
| (At 1st ANC visit) | | | |  | | |  |  |  | | |  | |  |  | |  | | |  |  |  | | |  |
|  |  |  |  |  | | |  |  |  | | |  | |  |  | |  | | |  |  |  | | |  |
| 2.3 Serious diseases | | | |  | | | 1 Diabetes | |  | | | 6 | | Anaemia | | |  | | | 11 | Tuberculosis |  | | |  |
|  | |  | | 2 | | | | Hypertension |  | | | 7 | | Gynaecological disease | | | | | | 12 | Sickle cell |  | | |  |
|  | |  | | 3 | | | | Heart diseases |  | | | 8 | | Liver disease | | |  | | | 13 | Others, specify: |  | | |  |
|  | |  | | 4 | | | | Epilepsy |  | | | 9 | | Kidney disease | | |  | | |  |  |  | | |  |
|  | |  | |  |  |  |  |  |  | | |  |  |  |  |  |  | | |  |  |  | | |  |
|  | |  | | 5 | | | | Malaria |  | | | 10 Lung disease | | | | |  | | |  |  | |  |  |  |
|  | |  | |  |  |  |  |  |  | | |  |  |  |  |  |  | | |  |  | |  |  |  |
| 2.4 Have you ever practised family planning: | | | | | | | | | 1 Yes | | |  | |  |  | |  | | |  |  |  | | |  |
|  | |  | |  | | |  |  | 2 No | | |  | |  |  | |  | | |  |  |  | | |  |
| 2.5 Antenatal care in this pregnancy: | | | | | | | | | 1 Yes | | | If yes: First medical appointment date: | | | | | | | | | |  | | |  |
|  | |  | |  | | |  |  | 2 No | | |  | |  |  | |  | | |  |  |  | | |  |
|  | |  | |  | | |  |  |  |  |  |  | |  |  | |  | | |  |  |  | | |  |
|  | |  | |  | | |  |  |  | | |  | | |  | |  | | |  | |  | | |  |
| 2.6 Number of visits: | | | |  | | |  |  |  | | | If date unknown, estimate | | | | | | | | 1 0-12 weeks | |  | | |  |
|  |  |  |  |  | | |  |  |  | | |  |  |  |  |  |  |  |  |  |  |  | | |  |
|  | |  | |  | | |  |  |  | | | first appointment: | | | | |  | | | 2 13-20 week | |  | | |  |
|  | |  | |  | | |  |  |  | | |  |  |  |  |  |  | | |  |  |  | | |  |
|  | |  | |  | | |  |  |  | | |  | |  |  | |  | | | 3 21-30 week | |  | | |  |
|  | |  | |  | | |  |  |  | | |  | |  |  | |  | | | 4 After 31 week | |  | | |  |
| **2.7 L.M.P** | |  | |  | | |  |  | Ultrasound | | | 1 | | Yes | | | **E.D.D** | | |  |  |  | | |  |
|  | |  | |  | | |  |  |  | | | 2 No | | | | |  | | |  |  | |  |  |  |
|  | |  | |  | | |  |  |  | | |  |  |  |  |  |  | | |  |  | |  |  |  |
| 2.8 Do you smoke | | | | Yes | | | |  | If yes, how many | | |  | |  |  | |  | | |  |  | |  |  |  |
|  |  |  |  |  |  |  |  |  |  |  |  |  | |  |  | |  | | |  |  | |  |  |  |
|  | |  | | No | | | |  | cigarettes per day: | | | | |  |  | |  | | |  |  | |  |  |  |
|  | |  | |  |  |  |  |  |  |  |  |  |  |  |  | |  | | |  |  | |  |  |  |
| 2.9 Do you drink alcoholic | | | |  | | | 1 Yes | | If yes: | | | 1 | | Every day | | |  | | |  |  |  | | |  |
| everages | | | |  | | | 2 No | |  | | | 2 More than once a week | | | | | | | |  |  |  | | |  |
|  | |  | |  | | |  |  |  | | | 3 Once a week | | | | |  | | |  |  |  | | |  |
|  | |  | |  | | |  |  |  | | | 4 | | Occasionally | | |  | | |  |  |  | | |  |
| 2.10 Diseases and | | | |  | | | 1 Yes (Specify below) | | | | |  | |  |  | |  | | |  |  |  | | |  |
| mplications during | | | |  | | | 2 No | |  | | |  | |  |  | |  | | |  |  |  | | |  |
| present pregnancy, | | | |  | | |  |  |  | | |  | |  |  | |  | | |  |  |  | | |  |
| including accidents: | | | |  | | | 1 Gestational diabetes | | | | | 7 | | Epilepsy | | |  | | | 14 | Gynaecological |  | | |  |
|  | |  | |  | | | 2 Diabetes | |  | | | 8 Bleeding | | | | |  | | | 15 Thromboembolic | |  | | |  |
|  | |  | |  | | | 3 Hypertension | |  | | | 9 Anaemia | | | | |  | | | 16 Heart disease | |  | | |  |
|  | |  | | 4 | | | | Preeclampsia, mild | | | | 10 Hyperemesis | | | | |  | | | 17 | Tuberculosis |  | | |  |
|  | |  | | 5 | | | | Preeclampsia, severe | | | | 11 Malaria | | | | |  | | | 18 | Lung disease |  | | |  |
|  | |  | | 6 | | | | Eclampsia |  | | | 12 Jaundice | | | | |  | | | 19 | Infections |  | | |  |
|  | |  | |  | | |  |  |  | | | 13 Schistosomiasis | | | | |  | | | 20 | Others, specify: |  | | |  |
|  | |  | |  | | |  |  |  | | |  | |  |  | |  | | |  |  |  | | |  |
| 3.1 | At birth | |  | | 1 | Singleton | | | | If multiple, add |  | |  | | | **3.2 Complications** | | | 1 PROM | | |  | | | |
|  |  |  |  | |  |  |  |  |  |  |  | |  | | |  |  |  |  |  |  |  | | | |
|  |  | |  | |  |  | | | |  |  | |  | | |  | | |  | | |  | | | |
|  |  | |  | | 2 | Multiple birth | | | | no. of children: | | |  | | | **during delivery:** | | | 2 Bleeding > 500ml | | | |  | |  |
|  |  | |  | |  |  |  | |  |  |  | |  | | |  | |  | 3 3-4 degree tear | | |  | | | |
| **3.3 Blood loss** (ml) | | |  | |  |  |  | |  | 3.4 Induction of | | | | | | 1 Yes | |  | 4 Abruption placenta | | |  | | | |
|  |  |  |  | |  |  |  | |  |  |  |  |  |  |  |  |  |  |  |  |  |  | | | |
|  |  | |  | |  |  |  | |  | labour | | |  | | | 2 No | |  | 5 Placenta Previa | | |  | | | |
|  |  | |  | |  |  |  | |  |  |  |  |  | | |  |  |  |  |  |  |  | | | |
|  |  | |  | |  |  |  | |  |  |  | |  | | |  | |  | 6 Other complications | | |  | | | |
| 3.5 | Gestational age at birth | | | |  |  |  | |  | 3.6 Mother's health after | | | | | | | | 1 Good |  | | |  | | | |
|  |  |  |  |  |  |  |  | |  |  |  |  |  |  |  |  |  |  |  | | |  | | | |
| clinical estimate | | |  | |  |  |  | |  | delivery | | |  | | |  | | 2 Fair |  | | |  | | | |
|  |  |  |  | |  |  |  | |  |  |  |  |  | | |  | |  |  | | |  | | | |
|  |  | |  | |  |  |  | |  |  |  | |  | | |  | | 3 Bad |  | | |  | | | |
|  |  | |  | |  |  |  | |  |  |  | |  | | |  | | 4 Maternal death | | | | |  | |  |

| **4 Status of the child** | | |  |  |  |  |  |  |  |  |  |  |  | | |
| --- | --- | --- | --- | --- | --- | --- | --- | --- | --- | --- | --- | --- | --- | --- | --- |
| 4.1 | Date of delivery: |  |  |  |  |  | 4.2 Birth weight (gram) | | | |  |  | |  |  |
|  |  |  |  |  |  |  |  |  |  |  |  |  | |  |  |
| 4.3 | Length (cm) |  |  |  |  |  | 4.4 Head circumference: | | | |  |  | |  |  |
|  |  |  |  |  |  |  |  |  |  |  |  |  | |  |  |
|  |  |  |  |  |  |  |  |  |  |  |  |  | |  |  |
|  |  |  |  |  |  |  |  |  |  |  |  |  |  | | |
| 4.5 | Presentation |  | 1 | Cephalic | | | 4.6 Status | | 1 Live born |  |  |  |  | | |
|  |  |  | 2 | Breech | | |  |  | 2 Live born transferred to Paediatric dept. | | | |  | | |
|  |  |  | 3 | Transverse | | |  |  | 3 Stillborn |  | **Cause** |  |  | | |
|  |  |  |  |  |  |  |  |  |  |  |  |  |  | | |
|  |  |  | 4 Other | | | |  |  | 4 Neonatal death | | **death** |  |  | | |
| 4.7 | If stillborn: |  | 1 | Dead before labour | | | **If stillborn also:** | | | 1 Dead before admission | | |  | | |
|  |  |  | 2 Dead during labour | | | |  |  |  | 2 Dead after admission | | |  | | |
|  |  |  | 3 Unknown, unspec | | | | **And** | | 1 Fresh |  |  |  |  | | |
|  |  |  |  |  |  |  |  |  | 2 Macerated | |  |  |  | | |
| **4.8 Apgar** | | **1 min** |  |  |  |  | **5 min** |  |  |  | **10 min** |  |  | | |
|  |  |  |  |  |  |  |  |  |  |  |  |  |  | | |
| **score** | |  |  |  |  |  |  |  |  |  |  |  |  | | |
|  |  |  |  |  |  |  |  |  |  |  |  |  |  | | |
| 4.9 | Mode of delivery: |  | 1 | Spontaneous | | | 4 CS elective | | | **Indication when** | | Primary: |  | | |
|  |  |  | 2 Vacuum, vaginal | | | | 5 CS others | | | **Caesarean section** | |  | |  |  |
|  |  |  | 3 | Forceps, vaginal | | | 6 Assisted breech | | |  |  | Secondary: |  | | |
|  |  |  |  |  |  |  | 7 Destructive operative | | | |  |  |  | | |
| 4.10 Does the child have | | |  |  | 1 Birth defects | |  |  |  |  |  |  |  | | |
|  |  |  |  |  |  |  |  |  |  |  |  |  |  | | |
| any of these conditions? | | |  |  | 2 Injuries | |  |  |  |  |  |  |  | | |
|  |  |  |  |  | 3 Diseases | |  |  |  |  |  |  |  | | |
|  |  |  |  |  | 4 HIV Positive | |  |  |  |  |  |  |  | | |
